# Winter Cough

**Published:** 1889-12-28

**Authors:** 


### The Practitioner's Outlook and Retrospect.

[*The Editor will be glad to receive offers of co-operation and contributions from members of the profession. There is no doubt that much valuable material full of interest and instruction is at present lost to science. This is largely so in the case of* (1) *the younger and less-known members of the hospital staff,* (2) *those connected with cottage hospitals, and* (3) *the great body of medical practitioners not attached to any institution. The co-operation of all is cordially invited to enable the Editor to make* The Hospital *of the utmost possible use and value to the profession. Specialists willing to review books on their special subjects are invited to communicate this fact at once. All letters should be addressed to* The Editor, The Lodge, Porchester Square, London, W.]

### MEDICINE.

### WINTER COUGH.

Winter cough is certainly not a very scientific term, but it is one very much in vogue. Popularly, the term is applied to any cough which is most troublesome in the winter, irrespective of what condition the cough depends upon, and cough may depend on a great number of conditions. In our view it would be better if the term could be restricted to that irritability of the parts about the throat which occurs to some persons whenever the colder weather sets in. But as it is, the asthmatic, the sufferer from bronchial irritation, the person with a long uvula, the dyspeptic, and the individual with a chronic nasal discharge, all speak of their "winter cough." Dr. William Murrell, lecturer on therapeutics, pharmacology, and materia medica at the Westminster Hospital, recommends chloride of ammonium and the Vereker inhaler in the *Medical Press* for November 6th, 1889. The inhaler consists essentially of three bottles with connecting tubes. In the first is placed hydrochloric acid; in the second a mixture of ammonia and water; whilst the third, which communicates with the other two by a three-way tube, acts as a wash bottle, and separates any trace of free acid or ammonia from the vapour. Several cases are recorded where the use of the inhaler either cured or very much benefited winter cough. A gentleman, "an M.P. by trade," was cured of constant sneezing and running from the eyes. A retired general was cured of deafness. By-the-bye, this general had consulted many specialists, and told Dr. Murrell that the advantage of consulting many doctors is that no two of them agree, "so you are not alarmed by what they tell you!" An architectural sculptor was also much benefited, his complaint being winter cough of some duration. There would seem, however, to be some little difficulty in managing the Vereker inhaler. For although Dr. Murrell states "the whole apparatus is perfectly simple," he devotes some space to telling us how to use it. But the difficulty is not always with the apparatus, but oftentimes with the patient, as "some patients do not know how to breathe, but take little puffs which are no good," but a few minutes' instruction and a practical illustration or two usually sets this right. Terebene, oil of cubebs, oil of sandal wood, and various other volatile drugs may be used in the inhaler.

